# miR-150-5p affects AS plaque with ASMC proliferation and migration by STAT1

**DOI:** 10.1515/med-2021-0357

**Published:** 2021-11-03

**Authors:** Yuan Bian, Wenqiang Cai, Hongying Lu, Shuhong Tang, Keqin Yang, Yan Tan

**Affiliations:** Department of Neurosurgery, Guigang City People’s Hospital, Guigang, 537100, China; Department of Neurosurgery, Guigang City People’s Hospital, No. 1, Zhongshan Middle Road, Guigang, 537100, China

**Keywords:** atherosclerosis, miR-150-5p, plaque stability, collagen metabolism, signal transducer and activator of transcription 1

## Abstract

We explore miR‐150‐5p in atherosclerosis (AS). The AS model was constructed using Apo E^−/−^ mice with an injection of the miR-150-5p mimic or an inhibitor. Pathological characteristics were assessed using Oil red O staining and Masson staining. Quantitative reverse transcription-polymerase chain reaction (qRT-PCR) and Western blot were used to analyze the expressions of microRNA-150-5p (miR-150-5p), STAT1, α-SMA (α-smooth muscle actin) and proliferating cell nuclear antigen (PCNA). Targetscan and dual-luciferase reporter assay were used to analyze the interaction between miR-150-5p and STAT1. The viability, migration, cell cycle and α-SMA and PCNA expressions in oxidized low-density lipoprotein (ox-LDL)-stimulated primary human aortic smooth muscle cells (ASMCs) were assessed using molecular experiments. miR-150-5p was reduced in both AS mice and ox-LDL-stimulated human aortic smooth muscle cells but STAT1 had the opposite effect. The miR‐150‐5p inhibitor alleviated the increase of lipid plaque and reduced collagen accumulation in the aortas during AS. Upregulation of α-SMA and PCNA was reversed by miR-150-5p upregulation. STAT1 was targeted by miR‐150‐5p, and overexpressed miR-150-5p weakened the ox-LDL-induced increase of viability and migration abilities and blocked cell cycle in ASMCs, but overexpressed STAT1 blocked the effect of the miR‐150‐5p mimic. This paper demonstrates that miR-150-5p has potential as a therapeutic target in AS, with plaque stabilization by regulating ASMC proliferation and migration via STAT1.

## 1 Introduction

Atherosclerosis (AS) is the main cause of cardiovascular and cerebrovascular diseases [1]. The progression of typical AS can be divided into four stages: the first stage is the initiation stage, namely endothelial activation and injury; the second stage is the occurrence stage, that is, the deposition of lipid under the intima and the formation of foam cells. The third stage is progressive, i.e., enhanced biological actions of vascular smooth muscle cells (VSMCs), enlargement of necrotic core in plaque, angiogenesis, etc. The final stage is termination, in which an unstable plaque ruptures and triggers an acute coronary event [2,3]. The above pathological process mainly involves the dysfunction of endothelial cells, VSMCs and macrophages [4]. Apoptosis of VSMCs is an important factor during the occurrence and development of AS [5]. VSMCs are considered to be a protective factor for plaque-stabilizing properties in advanced lesions [5]. Besides, excessive proliferation and migration of VSMCs are other crucial events of AS [6]. However, the underlying mechanisms that modulate the proliferation and migration of VSMCs in AS remain largely undefined.

The Janus kinase (JAK)/transducer and activator of transcription (STAT) 1 signaling pathway is a discovered stress response pathway that is widely involved in various cell progressions [7]. STAT1 is considered to be a therapeutic target in pro-atherogenic signals in vascular diseases [8]. A previous study has noted that immunohistochemical staining of phosphorylated STAT1 in carotid atherosclerotic plaques of human late AS has clearly observed that VSMCs in lesions highly expressed phosphorylated STAT1 compared to healthy blood vessels [9]. A recent report has demonstrated that the proliferation and migration of VSMCs could be prevented via STAT1-Kruppel-like factor 4 (KLF4) activation [10]. As a biomarker of VSMCs, in the less-differentiated VSMCs, the content of α-SMA is minimal but opposite in differentiated and matured VSMCs [11]. α-SMA also can be a biomarker of cell proliferation [12]. Proliferating cell nuclear antigen (PCNA) existed only in normal proliferating cells, played an important role in the initiation of cell proliferation, and was a good indicator of cell proliferation status [13].

MicroRNAs (miRNAs) are short non-coding RNA molecules (21–23 nucleotides). They are widely discovered in various organisms and participate in gene expression regulation at the post-transcriptional level [14]. Recently, studies have shown that miRNAs can be crucial regulators during the development of AS [15]. For instance, miR-221/222 regulate the proliferation and migration of VSMCs by interacting with P27 and P57 [16]. miR-150-5p was also found to be a tumor suppressor of cancers by inhibiting cell proliferation and migration [17,18]. In arthritis disease, endothelial proliferation, migration and tube formation can be suppressed by miR‐150‐5p [19]. Besides, mice with deleted miR-150 showed inhibition of inflammatory responses and a reduction of the AS lesion size [20]. Microvesicles containing miR‐150 secreted from patients with AS enhanced endothelial cell migration [21]. However, whether miR‐150‐5p also regulates the function of VSMCs to modulate the progression of AS remains rarely reported. Recent research indicates that lncRNA TNK2‐AS1 inhibits proliferation and migration of oxidized low-density lipoprotein (ox-LDL)-stimulated human aortic smooth muscle cells (HASMCs) [22]. Additionally, a recent report has shown that STAT1 is the downstream target of miR-150-5p [23]. However, the role of STAT1 and miR-150-5p in the regulation of VSMCs during AS has not been revealed.

To illustrate the function of miR‐150‐5p in AS, Apo E^−/−^ mice fed with high-fat food were injected with a miR‐150‐5p mimic or inhibitor; then, pathological analysis was then conducted to assess the influence of miR‐150‐5p on AS. Furthermore, the primary HASMCs were treated with ox-LDL as the *in vitro* model of AS [17], and then miR‐150‐5p and STAT1 were overexpressed in ox-LDL-stimulated HASMCs to assess the role of miR‐150‐5p and STAT1 in the dysfunction of VSMCs. We hoped to find a potential therapeutic target for AS based on the experiments performed in this research.

## 2 Methods

### 2.1 Animal model and treatment

Sixty C57BL/6J and Apo E^−/−^ mice (8 week-old, male, 18–22 g) were purchased from Beijing Vital River Laboratory Animal Technologies Co. Ltd. All mice were kept in a specified pathogen-free (SPF) room at 22–25°C with light–dark cycle (12 h:12 h). High-fat feed (0.25% cholesterol, 21% fat) was obtained from Beijing Keao Xieli Feed Co., Ltd. After 1 week of adaptive feeding in SPF grade environment, mice were divided into a model group (45 mice, Apo E^−/−^) and control group (15 mice, C57BL/6J). Mice in the model group were given a high-fat diet for 12 weeks, and mice in the control group were given a normal diet for 12 weeks. After 16 weeks, mice in the model group were further divided into three groups: Model + NC group, Model + M group, Model + I group, and 15 mice were in each group. The mice in the Model + NC group were injected with 5 × 10^8^ TU/mL lentivirus–negative control suspension (NC, 20 µL) for mimic and inhibitor via the tail vein. The mice in the Model + M group were injected with 5 × 10^8^ TU/mL lentivirus suspension (20 µL) containing the miR-150-5p mimic (5′-UCUCCCAACCCUUGUACCAGUG-3′) via the tail vein. The mice in the Model + I group were injected with 5 × 10^8^ TU/mL lentivirus suspension containing the miR-150-5p inhibitor (20 µL, 5′-CACUGGUACAAGGGUUGGGAGA-3′) via the tail vein. The C57BL/6 J mice in the control group were treated with isometric saline via the tail vein injection. Lentivirus delivery was repeated in 2 weeks. All sequences were purchased from Hanheng Biotechnology (Hanheng Biotechnology Co., Ltd., Shanghai, China).

### 2.2 Sample collection

The mice were anesthetized at the 4th week after the first lentivirus injection (8 mg/100 g ketamine + 2 mg/100 g xylazine + 0.06 mg/100 g atropine), and then perfused with PBS for 2 min with a needle inserted into the left ventricle. The aortic root vessels were separated and fixed in 4% paraformaldehyde at room temperature overnight and then embedded in an optimal cutting temperature (OCT) compound. The rest of the aortas were used for gene expression analysis.

### 2.3 Quantitative reverse transcription-polymerase chain reaction (qRT-PCR)

After the aortas were collected, or cells were collected, the total RNA from tissues or cells was extracted using the Trizol reagent (Invitrogen) in accordance with the manufacturer’s instructions. The mRNAs were reverse-transcribed into cDNA using a cDNA Synthesis Kit (cat: 6210B, Takara, Japan), and were then quantified by the use of SYBR green mix (A25742, Applied Biosystems, USA). The reaction parameters were set as follows: (the first step) 95°C, 5 min; (the second step) 40 cycles of 95°C, 15 s, 60°C for 30 s; (the last step) 70°C for 10 s. GAPDH was detected as an internal control. The 2^−ΔΔCT^   method was used for comparing the relative expression results[24]. The paired primers for miR-150-5p were: forward, 5′-AACCCTTGTACCAGTGGTCG-3′; reverse, 5′-GTATCCAGTGCGTGTCGTGG-3′. The paired primers for U6 snRNA were: forward, 5′-CTCGCTTCGGCAGCACA-3′; reverse, 5′-AACGCTTCACGAATTTGCGT-3′. The paired primers for STAT1 were: forward, 5′-TCACAGTGGTTCGAGCTTCAG-3′; reverse, 5′-CGAGACATCATAGGCAGCGTG-3′. U6 snRNA was used as the internal reference for normalization of miR-150-5p levels. The primers were designed and synthesized by the Sangon Company (Sangon Biotech, Shanghai, China).

### 2.4 Oil red O staining

After anesthetized blood collection and euthanasia of the mice, the chest and abdomen of the mice were cut open, and the remaining tissues were cut off to fully expose the aortic vessels. The brachiocephalic artery was then removed and fixed in 4% paraformaldehyde for 2 h at room temperature. Then, the tissue was dehydrated overnight at 4°C in 30% sucrose. After that, the tissues were used to make a frozen section (6 µm). Then, the frozen section was stained with Oil red O stain solution for 10 min at 60°C. Afterward, the section was washed with 1% HCl – alcohol for 5 s and water for 2 min. After drying at room temperature, glycerine-gelatin was added to seal the plaque, and the expression of plaque was observed under an optical microscope (TS100, Nikon, Japan).

### 2.5 Masson staining

The Masson’s Trichrome Stain Kit (G1340-100, Solarbio, China) was purchased for staining. The frozen sections (6 µm) were also used for Masson staining. Bouin solution was used to fix the sections at 37°C for 2 h. Then, the sections were washed with water and then stained with hematoxylin for 6 min at room temperature. Next, the Masson solution was added onto the sections for 5 min at room temperature. Then, one drop of phosphomolybdic acid was added into the sections for 5 min at room temperature followed by staining with one drop of light green for 5 min. Afterward, the section was soaked in 0.2% acetic acid solution and subjected to dehydration processing. Finally, xylene was used to remove the grease and the sections were sealed with neutral resin. The morphology was observed under an optical microscope (TS100, Nikon, Japan).

### 2.6 Western blot

The aorta tissues were collected and homogenized in 200 µL of RIPA lysis buffer (P0013C, Beyotime, China) on ice. The aorta specimen was further lysed using ultrasound for 2 min and the interval was 5 s at 4°C. Next, the lysate was collected by centrifugation at 13,000×*g*/min for 10 min. For protein extraction from cells, HASMCs were treated with 200 µL of RIPA lysis buffer on ice and the lysate was collected, and the protein content was obtained by centrifugation at 13,000×*g*/min for 10 min. After that, the BCA method was used to quantify the protein.

Then, 20 µg of protein was separated using SDS-PAGE. After that, the protein was transferred to PVDF membranes (FFP24, Beyotime, China). Then, 5 mL of 5% skim milk was used to block the membranes. Afterward, the primary antibodies were incubated with the membranes overnight at 4°C followed by the secondary antibodies incubation at room temperature for 2 h. Finally, the expression of the related proteins was observed and detected under the gel imaging system (iBright™ CL750, cat: A44116, Invitrogen, Carlsbad, CA, USA). GAPDH was used as the internal control. The primary antibodies were as follows: STAT1 (ab3987, 91 kD, Abcam, USA), α-SMA(ab5694, 42 kD, Abcam, USA), PCNA (ab92552, 29 kD, Abcam, USA) and GAPDH (ab8245,36kD, Abcam, USA).

### 2.7 Targetscan analysis

Targetscan Mouse (http://www.targetscan.org/mmu_72/) was used to analyze the potential interaction between miR-150-5p and STAT1.

### 2.8 Cell culture and ox-LDL treatment

HASMCs (cat: PCS-100-012) were obtained from ATCC, USA. The smooth muscle cell growth medium containing 10% FBS (Thermo Fisher Scientific, Waltham, USA) was used to maintain HASMCs at 37°C with 5% CO_2_. Ox-LDL was purchased from Sigma-Aldrich (St. Louis, USA) and the final concentrations for the HASMC treatment were 50 µg/mL, and the incubation time was set as 24 h.

### 2.9 Dual-luciferase reporter assay

The fragment of STAT1 containing the wild type (WT) or mutant (MUT) binding site for miR-150-5p was subcloned into the pmirGLO luciferase reporter vector (E1330, Promega, Madison, WI, USA). HASMCs were co-transfected with 0.1 µg of a recombinant plasmid containing STAT1-WT vector or STAT1-MUT vector with or without the miR-150-5p mimic (3 pmol) using Lipofectamine 2000 (cat: 11668019, Invitrogen, Carlsbad, CA, USA). After 48 h incubation at 37°C, the luciferase activity of HASMCs was determined with a Luciferase Reporter Assay kit (E1910, Promega, Madison, WI, USA). Finally, a microplate luminometer (11300010, Berthold, Germany) was used to detect the firefly luciferase signal and renilla luciferase signal. The relative luciferase activity was determined as the ratio of firefly luciferase signal to renilla luciferase signal.

### 2.10 Cell transfection

Lipofectamine 2000 (cat: 11668019, Invitrogen, Carlsbad, CA, USA) was used for cell transfection. First, ox-LDL-stimulated HASMCs were grown in a 6-well plate. Then, 2 μg of overexpressed STAT1 plasmid (the recombinant pcDNA 3.1 containing the cDNA of STAT1), or 100 pmol of the miR-150-5p mimic or mimic control was mixed into the 8 μL of Lipofectamine 2000 reagent for 15 min at room temperature. Then, the mixture was added to the well and cells were further cultured for 24 h at 37°C. Finally, cells were collected for further experiments.

### 2.11 Cell viability analysis

The CCK-8 kit (cat: C0038, Beyotime, China) was used to assess the viability of HASMCs. In brief, the treated HASMCs (1 × 10^4^ cells/well, 100 μL) were grown in a 96-well plate. At the indicated time, 10 µL of CCK-8 was incubated with the cells for 4 h at 37°C away from light. Then, the absorbance (450 nm) was detected using a Microplate Reader (PLUS 384, Molecular Devices, USA).

### 2.12 Cell cycle

The transfected cells in the logarithmic growth phase were used to inoculate a 24-well plate at 1 × 10^6^ cells/mL with 1 mL. After the cells were centrifuged at 800×*g* for 5 min, they were washed twice with precooled PBS; then precooled 75% ethanol was added to the cells and were fixed at 4°C for more than 4 h. Then, 400 μL of the PI solution was used to stain the cells and incubated at 4°C in the dark for 30 min. Finally, flow cytometry (Beckman Coulter, CA, USA) was used to detect the cell cycle distribution.

### 2.13 Migration analysis

The migration ability was determined using wound healing. Briefly, HASMCs were seeded into the 6-well plate. When cells reached 100% confluence, a scratch was made using a 10 μL pipette tip. Then, PBS was used to wash the floating cells. After culturing in a serum-free medium for 48 h, cell migration was observed. The relative migration distance was calculated as follows: (the width at 0 h-the width at 48 h)/the width at 0 h*100%. Three fields were selected for each group.

### 2.14 Statistical analysis

Data were displayed as mean ± standard deviation (SD). The difference between the two groups was analyzed using Student’s *t*-test. The difference among more than two groups was determined using one-way ANOVA followed by Tukey’s post-hoc test. We used SPSS v.19.0 software (IBM, Armonk, NY, USA) to conduct the statistical analysis and GraphPad Prism 5.02 software (La Jolla, CA, USA) to construct the graph. All experiments were repeated three times.


**Ethical statement:** Animal experiments were approved by the Ethics Committee of Guigang City People’s Hospital (No. 20190212A). All efforts were taken to minimize animal suffering.

## 3 Results

### 3.1 miR-150-5p regulated the plaque stability, collagen metabolism and proliferation biomarkers expressions in ApoE^−/−^ mice

**Figure 1:**
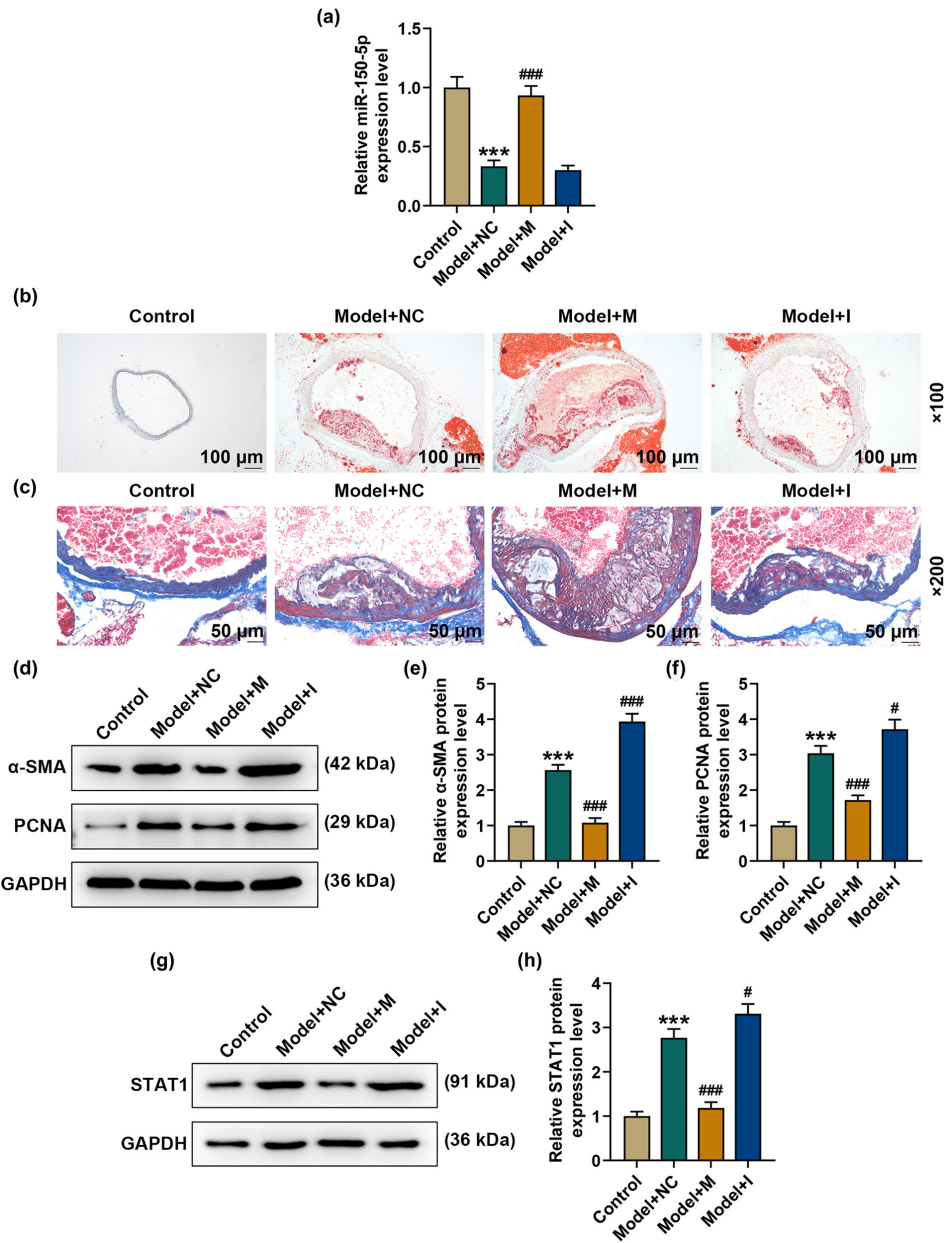
miR-150-5p regulated plaque stability, collagen metabolism and expressions of proliferation biomarkers in ApoE^−/−^ mice. (a) Transfection efficiency of the miR-150-5p mimic and inhibitor in the aortas of ApoE^−/−^ mice was detected using qPCR. U6 snRNA was used as the internal reference for normalization of miR-150-5p levels. (b) The level of lipid plaques in aortas of ApoE^−/−^ mice was determined by Oil red O staining. Magnification: 100×, scale bar = 100 μm. (c) Masson staining was performed to assess the collagen accumulation in aortas of ApoE^−/−^ mice. Magnification: 200×, scale bar = 50 μm. (d)–(h) Western blot analysis of α-SMA, PCNA and STAT1 expressions in aorta tissues. GAPDH was used as the internal reference. Three mice were in each group. ^***^ *p* < 0.001 vs control; ^#^ *p* < 0.05, ^###^ *p* < 0.001 vs Model + NC. NC: negative control for the mimic and inhibitor. M: miR-150-5p mimic. I: miR-150-5p inhibitor. All these experiments were performed three times independently. qRT-PCR: quantitative reverse transcription-polymerase chain reaction; ox-LDL: oxidized low-density lipoprotein; M: miR-150-5p mimic; NC: negative control (empty vector).

To investigate miR-150-5p in AS, an AS model was constructed in ApoE^−/−^ mice. qPCR analysis indicated that miR-150-5p was reduced in aortas of AS mice, while it was successfully overexpressed and silenced by the miR-150-5p mimic and inhibitor injection (*p* < 0.001, Figure 1a).

Next, to assess the influence of miR-150-5p on plaque stability and collagen metabolism in ApoE^−/−^ mice, the aorta frozen section was prepared. Oil red O staining of the aorta frozen section indicated that in the control group, the aortic intima was slightly thickened, and a small amount of lipid was deposited in the wall of the aorta; besides, a small amount of lipid plaque was stained red (Figure 1b). However, in the model group, the aortic intima was obviously thickened, lipid deposition in the wall was enhanced, and lipid plaques were stained red (Figure 1b). More importantly, the miR-150-5p inhibitor could weaken the pathological alterations in the model mice; on the contrary, the miR-150-5p mimic could aggravate the pathological alterations in the model mice with a significant increase of lipid plaque and a thickened aortic intima (Figure 1b). Masson staining was performed to assess the collagen accumulation in aortas of ApoE^−/−^ mice. The results showed that collagen accumulation (blue) was reduced in the model group while the reduction of collagen accumulation in aortas could be attenuated by the miR-150-5p inhibitor but was further enhanced by the miR-150-5p mimic (Figure 1c).

To further confirm the effect of miR-150-5p on proliferation, Western blot analysis of proliferation biomarker (α-SMA and PCNA) expressions in aorta tissues was performed [25]. Data indicated that α-SMA and PCNA were promoted in the model group, while the miR-150-5p mimic blocked the increase of α-SMA and PCNA in aortas; on the contrary, the miR-150-5p inhibitor enhanced the expression of α-SMA and PCNA (*p* < 0.05, Figure 1d–f). We also discovered that STAT1 was upregulated in the model group, and, more interestingly, we found that the upregulation of STAT1 in the model group could be weakened by miR-150-5p upregulation but enhanced by the miR-150-5p inhibitor (*p* < 0.05, Figure 1g–h).

### 3.2 miR-150-5p regulated the expression of STAT1 in ox-LDL-stimulated HASMCs

**Figure 2:**
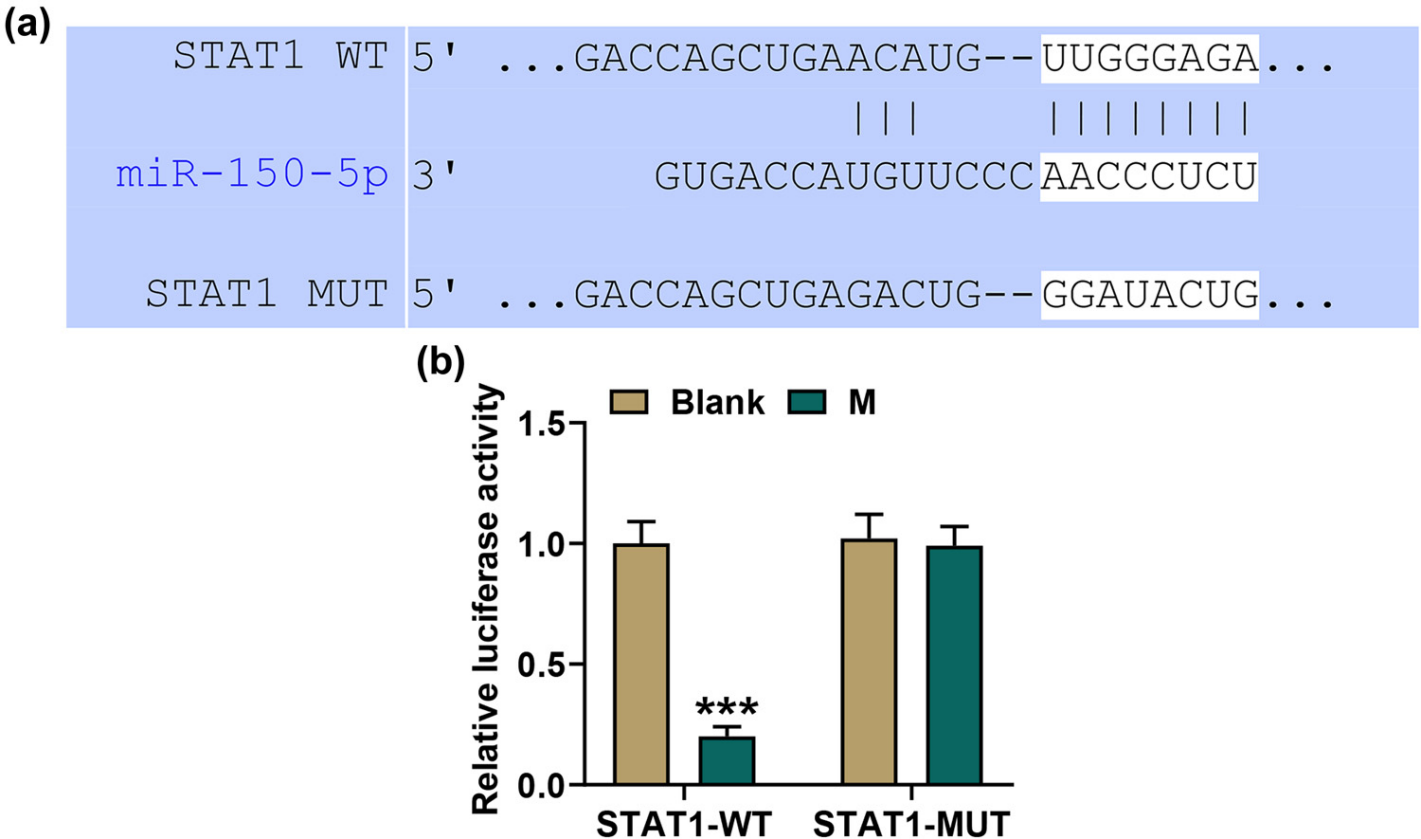
STAT1 was a direct target gene of miR-150-5p. (a) Targetscan predicted that miR-150-5p might bind to STAT1 mRNA. (b) The interaction between miR-150-5p and STAT1 was confirmed by using a dual-luciferase reporter assay. HASMCs were co-transfected with the recombinant plasmid containing the STAT1 WT sequence (WT) or mutant sequence (MUT) with or without the miR-150-5p mimic. M: miR-150-5p mimic; Blank: cells were treated with PBS without transfection. ^***^ *p* < 0.001 vs Blank. The luciferase reporter assay was repeated three times.

To explore the relationship between STAT1 and miR-150-5p, Targetscan was used to discover the potential interaction between STAT1 and miR-150-5p. As shown in Figure 2a, a binding site for miR-150-5p was present in STAT1 mRNA. Further analysis confirmed that STAT1 was a direct target gene of miR-150-5p. Dual-luciferase reporter assay showed that miR-150-5p upregulation suppressed the luciferase activity of the recombinant plasmid containing STAT1 WT (*p* < 0.001), while miR-150-5p upregulation had no effect on the luciferase activity of the STAT1 MUT recombinant plasmid (Figure 2b).

**Figure 3:**
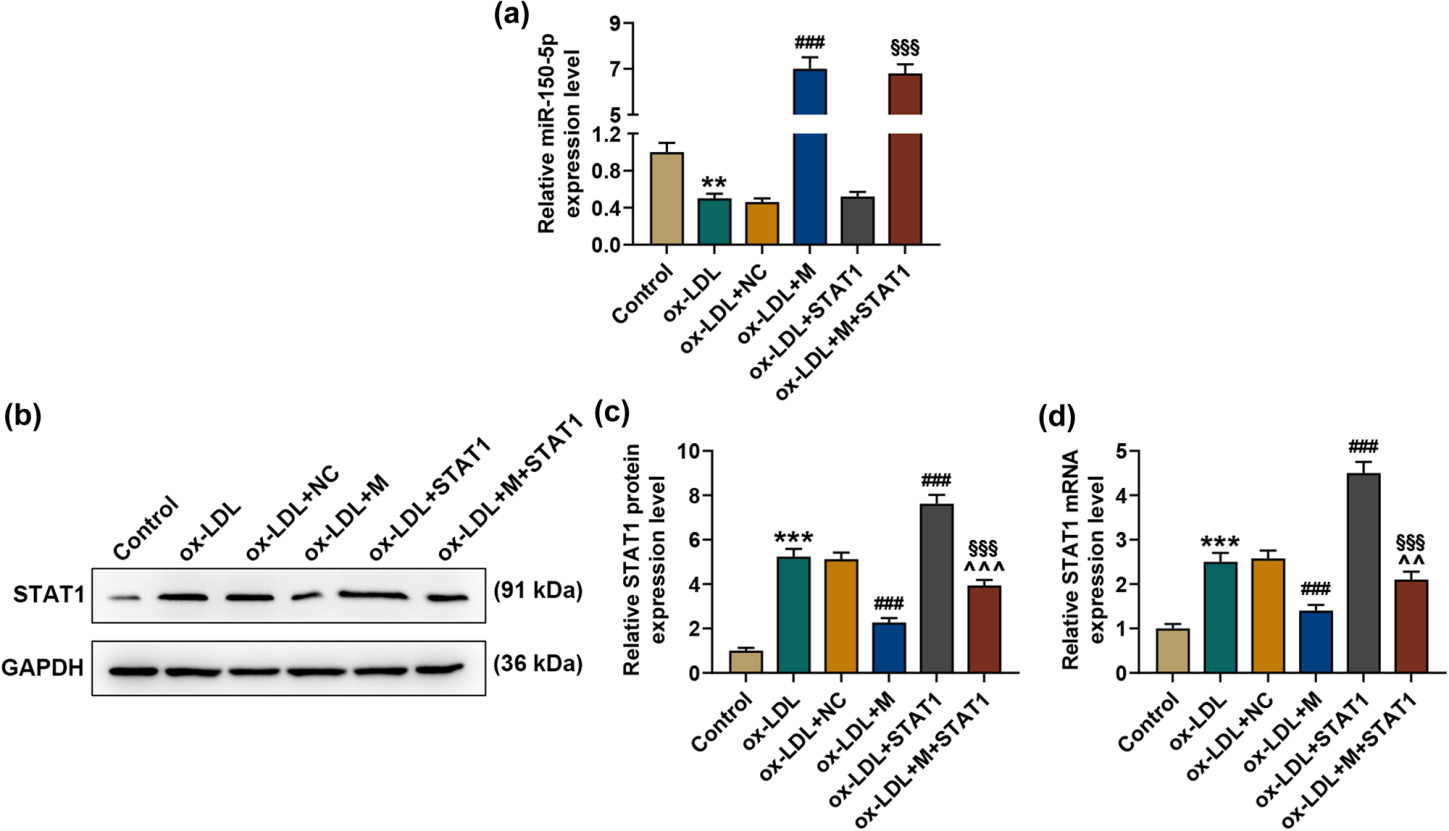
miR-150-5p regulated the expression of STAT1 in ox-LDL-stimulated HASMCs. (a) The relative expression of miR-150-5p was detected by qRT-PCR. U6 snRNA was used as the internal reference for normalization of miR-150-5p levels. (b)–(d) The relative expression of STAT1 was detected by qRT-PCR and Western blot. GAPDH was used as the internal reference. ^**^ *p* < 0.01,^***^ *p* < 0.001 vs Control; ^###^ *p* < 0.001 vs ox-LDL ^+^ NC; ^^^^ *p* < 0.01, ^^^^^ *p* < 0.001 vs ox-LDL + M; ^§§§^ *p* < 0.001 vs ox-LDL + STAT1. qRT-PCR: quantitative reverse transcription-polymerase chain reaction; ox-LDL: oxidized low-density lipoprotein; M: miR-150-5p mimic; NC: negative control (empty vector). All these experiments were performed three times independently.

To further verify the interaction between STAT1 and miR-150-5p, ox-LDL-stimulated HASMCs were constructed. qPCR analysis showed that miR-150-5p was reduced in ox-LDL-treated HASMCs, while miR-150-5p upregulation could increase the level of miR-150-5p (*p* < 0.001, Figure 3a). Besides, we also found that overexpressed STAT1 had no influence on miR-150-5p (Figure 3a). STAT1 expression was also assessed, and the results indicated that ox-LDL stimulation promoted the expression of STAT1, while miR-150-5p upregulation inhibited the expression of STAT1; besides, the increase of STAT1 induced by ox-LDL stimulation with overexpressed STAT1 could be weakened by miR-150-5p upregulation (*p* < 0.001, Figure 3b–d).

### 3.3 miR-150-5p influenced the viability, cell cycle, migration and expressions of proliferation biomarkers of ox-LDL-stimulated HASMCs via STAT1

**Figure 4:**
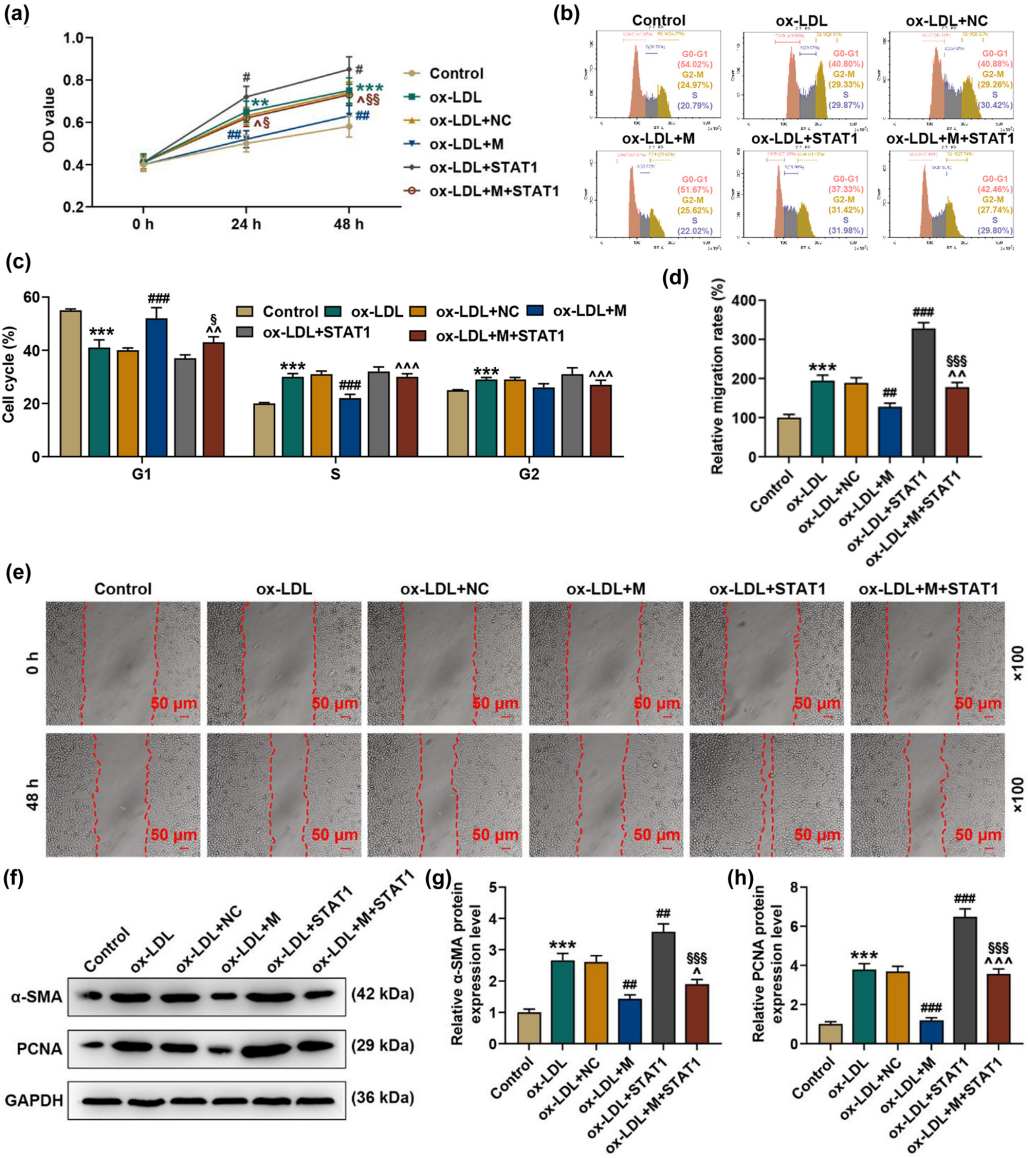
miR-150-5p influenced the viability, migration and expressions of proliferation biomarkers of ox-LDL-stimulated HASMCs via STAT1. (a) CCK-8 assay was used to determine the viability of ox-LDL-stimulated HASMCs transfected with the mimic or overexpressed STAT1 at 0 h, 24 h and 48 h. (b and c) Flow cytometry was used to detect the cell cycle of ox-LDL-stimulated HASMCs transfected with mimic or overexpressed STAT1. (d) The relative migration rate normalized to the control group was calculated. (e) Wound healing assay was performed to assess the cell migration ability. Magnification: 100×, scale bar = 50 μm. (f)–(h) Western blot analysis of α-SMA and PCNA in ox-LDL-stimulated HASMCs transfected with the mimic or overexpressed STAT1. GAPDH was used as the internal reference. ^**^ *p* < 0.01,^***^ *p* < 0.001 vs Control; ^#^ *p* < 0.05, ^##^ *p* < 0.01, ^###^ *p* < 0.001 vs ox-LDL + NC; ^^^ *p* < 0.05, ^^^^ *p* < 0.01, ^^^^^ *p* < 0.001 vs ox-LDL + mimic; ^§^ *p* < 0.05, ^§§^ *p* < 0.01, ^§§§^ *p* < 0.001 vs ox-LDL + STAT1. All these experiments were performed three times independently. OD: optical density; M: miR-150-5p mimic; NC: negative control (empty vector); ox-LDL: oxidized low-density lipoprotein; HASMCs: human aortic smooth muscle cells; α-SMA, α-smooth muscle actin (α-SMA); PCNA: proliferating cell nuclear antigen; STAT1: transducer and activator of transcription STAT 1.

Finally, we explored the function of miR-150-5p-STAT1 in ox-LDL-stimulated HASMCs. CCK-8 assay showed that overexpressed miR-150-5p inhibited the viability of HASMCs when induced with 100 g/mL ox-LDL, while overexpression STAT1 reverses the effect of overexpressed miR-150-5p (*p* < 0.001, Figure 4a). We used flow cytometry to detect the cell cycle, and the results showed that overexpression of miR-150-5p can reverse the effect of 100 μg/mL ox-LDL in inhibiting cycle arrest, while STAT1 reverses the effect of overexpression of miR-150-5p (*p* < 0.001, Figure 4b–c). Wound healing assay confirmed that ox-LDL-stimulated HASMCs showed an increase of migration rates, while that increase could be weakened by miR-150-5p upregulation but enhanced by overexpressed STAT1 (*p* < 0.05, Figure 4d–e). Further analysis of expressions of proliferation biomarkers by Western blot suggested that α-SMA and PCNA expressions were induced by ox-LDL stimulation but reduced by overexpressed miR-150-5p and promoted by STAT1 overexpression (*p* < 0.01, Figure 4f–h).

## 4 Discussion

In the present study, we found that miR-150-5p was reduced in both AS mice and HASMCs stimulated by ox‐LDL, a well‐documented risk contributor for AS, but STAT1 had an opposite pattern. miR-150-5p downregulation promoted plaque stabilization via increased smooth muscle cells and collagen content and decreased lipid accumulation in the aortas during AS. *In vitro* functional studies showed that overexpressed miR-150-5p attenuated the ox-LDL-induced increase in proliferation and migration of HASMCs by targeting STAT1. Collectively, our findings showed the potential role of miR-150-5p in regulating atherosclerotic plaque stability and in regulating proliferation and migration in the ox‐LDL‐stimulated cellular model of AS via targeting STAT1.

In recent years, there have been many studies on the preventive and therapeutic effects of miRNAs in the cardiovascular system [26,27]. Targeted delivery of miRNA has been developed for the AS treatment [28]. Increasing reports have demonstrated the important role of miR-150-5p in cardiovascular diseases (CVDs) [29,30,31,32]. Accumulation of lipid plaques in arterial is the main feature of AS [33]. In our research, we found that lipid plaques were obviously accumulated in the aortas of ApoE^−/−^ mice with AS. Collagens are the main constituents of the extracellular matrix and are involved in aneurysm formation and calcification [34]. Collagens can also provide tensile strength to the fibrous cap and protect against plaque rupture, a devastating complication of AS [35]. In this study, Masson staining showed that collagen accumulation in aortas of ApoE^−/−^ mice with AS was reduced, while the miR-150-5p inhibitor could obviously increase the collagen content. As a biomarker of VSMCs, in the less-differentiated VSMCs, the content of α-SMA is minimal, but opposite in differentiated and matured VSMCs [11]. α-SMA can also be a biomarker of cell proliferation [12]. PCNA existed only in normal proliferating cells and played an important role in the initiation of cell proliferation, and was a good indicator of cell proliferation status [13]. In this research, miR-150-5p downregulation could enhance the increase of α-SMA and PCNA expressions in the aortas of ApoE^−/−^ mice with AS, which was partly similar to the data in pulmonary artery smooth muscle cells [36]. These results taken together suggested that miR-150-5p downregulation might be a therapeutic strategy for AS treatment. Additionally, mice with double-knockout of miR-150^−/−^ and ApoE^−/−^ appeared to be of obvious atherosclerotic lesion size and stability could be reduced significantly, and macrophage and inflammatory response might be involved in this progression [20]. Our study found that miR-150-5p downregulation has a therapeutic function in AS mice, with plaque stabilization, via reducing the lipid accumulation and increasing the collagen deposition and smooth muscle cells.

STAT1 is widely expressed in multiple cells to promote cell proliferation and migration [37,38]. Consistent with a recent study [39], we found that STAT1 was upregulated in AS mice. The following bioinformatics analysis and luciferase assay further demonstrated that STAT1 could bind to miR-150-5p and interfere with its expression. Moreover, STAT1 expression was inversely regulated by miR-150-5p expression in ox‐LDL‐stimulated HVSMCs. In addition to increasing the thickness of the fibrous cap in plaques, VSMCs also promote macrophage apoptosis by reducing the size of the necrotic core [40]. Hence, we proceeded to explore whether the effects of STAT1 on proliferation and migration were mediated by miR-150-5p in ox-LDL-stimulated HVSMCs. The results indicated that miR-150-5p overexpression showed inhibitory effects in proliferation and migration of ox-LDL-stimulated HASMCs, which was consistent with Cai’s finding [22]. A recent study demonstrated that reduced STAT1 expression or decreased STAT1 activation may be effective approaches for reducing the incidence of AS [41]. However, in our study, STAT1 overexpression promoted proliferation and migration of ox-LDL-stimulated HVSMCs, which may be beneficial for atherosclerotic plaque stability. Additionally, STAT1 overexpression-mediated proliferation and migration effects were greatly reversed by miR-150-5p overexpression in ox-LDL-stimulated HVSMCs.

Inflammation plays an important role in the host’s defense against infectious pathogens and injuries but it also contributes to AS pathophysiology. A previous review has shown the STAT1-target gene as promising markers of vascular inflammation, and STAT1 as a potential target for the development of new immunosuppressive and anti-inflammatory agents for the treatment of CVDs [42]. Additionally, STAT1 has been regarded as a point of convergence for the cross-talk between the pro-atherogenic interferon (IFN)-γ, toll-like receptors and interleukin-6-activated pathways in immune and vascular cells, thus amplifying pro-inflammatory signals, which augments migration of ASMCs and leukocytes, leukocytes to endothelial cell adhesion and foam cell formation [8]. Moreover, miR-150 deficiency inhibits AS progression and inflammatory factor secretion [20]. Thus, in the future, exploring the role of STAT1 and miR-150-5p in inflammation during AS may encompass a novel mechanism involved in the occurrence and development of AS.

Collectively, miR-150-5p downregulation could attenuate AS development in AS mice by promoting plaque stabilization via the reduction of lipid accumulation and the increase of collagen deposition and smooth muscle cells. Moreover, miR-150-5p exerted its effects on inhibiting HVSMC proliferation and migration by targeting STAT1. The present study revealed miR-150-5p and STAT1 in regulating atherosclerotic plaque stability and provided an underlying mechanism for miR-150-5p-based therapeutic prevention of AS.
